# Design and Computational Modeling of a Modular, Compliant Robotic Assembly for Human Lumbar Unit and Spinal Cord Assistance

**DOI:** 10.1038/s41598-017-14220-3

**Published:** 2017-10-31

**Authors:** Gunjan Agarwal, Matthew A. Robertson, Harshal Sonar, Jamie Paik

**Affiliations:** Ecole Polytechnique Federale de Lausanne (Swiss Federal Institute of Technology, EPFL) Reconfigurable Robotics Laboratory, EPFL-IGM-RRL, MED 11326, Station 9, CH-1015 Lausanne, Switzerland

## Abstract

Wearable soft robotic systems are enabling safer human-robot interaction and are proving to be instrumental for biomedical rehabilitation. In this manuscript, we propose a novel, modular, wearable robotic device for human (lumbar) spine assistance that is developed using vacuum driven, soft pneumatic actuators (V-SPA). The actuators can handle large, repetitive loads efficiently under compression. Computational models to capture the complex non-linear mechanical behavior of individual actuator modules and the integrated assistive device are developed using the finite element method (FEM). The models presented can predict system behavior at large values of mechanical deformations and allow for rapid design iterations. It is shown that a single actuator module can be used to obtain a variety of different motion and force profiles and yield multiple degrees of freedom (DOF) depending on the module loading conditions, resulting in high system versatility and adaptability, and efficient replication of the targeted motion range for the human spinal cord. The efficacy of the finite element model is first validated for a single module using experimental results that include free displacement and blocked-forces. These results are then extended to encompass an extensive investigation of bio-mechanical performance requirements from the module assembly for the human spine-assistive device proposed.

## Introduction

Spinal cord injury (SCI) resulting from a traumatic movement leads to a deformation of the neural and vascular structure of the spinal cord^1^. In addition to accidental SCI cases, clinical observations have indicated that the physiological changes in comprising soft tissues throughout human lifetime lead to an overall reduction in the available space for the neural structures (spinal cord, medullar cone and roots) and that this effect is amplified by vertebrae slip due to the weakness of vertebral facets, ligamentous ossification, vertebral joint fusion and other factors such as osteoporosis. The biomechanics of different components of the spinal column, including the spinal cord and the vertebrae, is fairly well described and quantified in literature including computational studies using FEA^2,3^. However, solutions to help restore the lost mechanical strength and functionalities of the lumbar unit and the spinal cord even in part, following the injury situations described above, and enabling the overall mobility and high flexibility for the patient are still lacking. In the cases of lack of spinal mobility, along with several other scenarios, such as one where a patient may be suffering from a stroke in which cases repetitive motions have been reported to be highly beneficial^4^ in order to restore normal motor control, an artificial wearable device for spine assistance can be very desirable.

**Figure 1:**
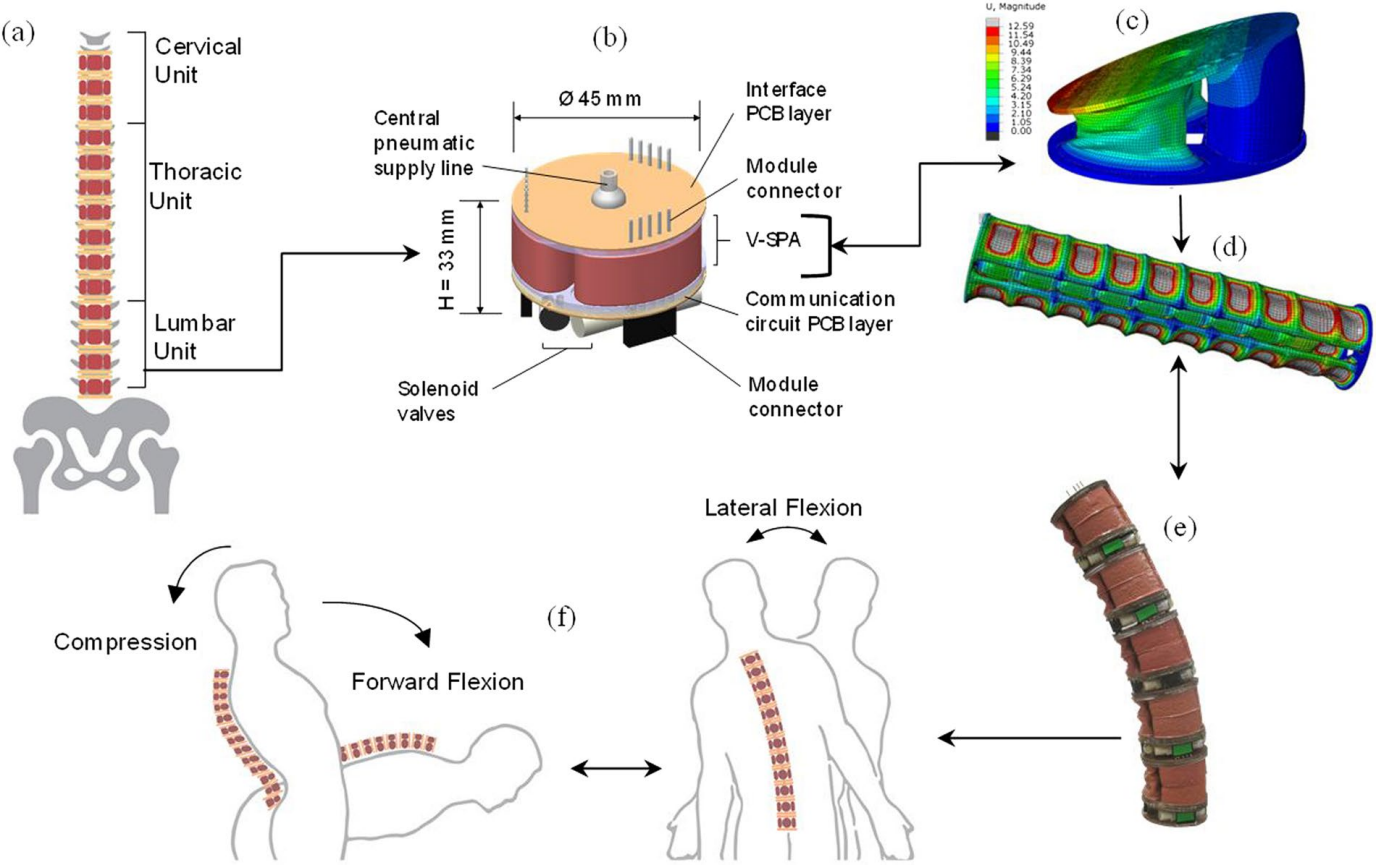
**(a)** Human spinal column representation comprising of the lumbar unit consisting of the vertebrae, intervertebral discs, spinal cord and other components. **(b)** Representation of the spinal column unit with a vacuum driven soft pneumatic actuator (V-SPA) module. **(c)** Numerical simulation results for a single V-SPA module. **(d)** Simulation results for a pressurized V-SPA module assembly with fixed ends. **(e)** V-SPA assembly undergoing free bending displacement. **(f)** Schematic representation of replication and assistance of spinal column motion with the help of the robotic assembly.

The functional building blocks and key system structural requirements required to achieve such an assistive system are presented in this work (Fig. 1), after analyzing mechanically related pathologies of the lumbar unit and the spinal structure by observing spinal cord deformations under different loading scenarios^2^. By studying the localization and the magnitude of maximum equivalent stress and shear stresses on the lumbar unit and the spinal cord, design requirements are elaborated for the assistive wearable device proposed. Physical properties of the vertebrae, ligaments, intervertebral discs, and the spinal cord are taken into account under loading such as compression, and combined loading, flexion and lateral bending to evaluate the pressure undergone by different components of the lumbar unit. Since the primary mode of mechanical failure is in compressive loading, high compression withstanding capabilities are desired from the proposed system, in order to replicate and efficiently assist with the diminished functionalities of the injured spinal column.

Soft materials and systems are light weight, highly flexible and adaptable, and inherently safer, making them better suited for building such systems as compared to their rigid counterparts. Due to these advantages, soft robotics^5–12^ has recently emerged as a dynamically evolving field, to create novel systems with pre-programmed and predictable capabilities out of elastomeric materials, capable of withstanding large deformations. Apart from applications in diverse fields, ranging from bioinspired and biomimetic systems^6–8^, adaptable locomotion on unstructured terrains^9^ and operating surgical tools^10^, soft robotic systems and structures are particularly well-suited for applications in biomedical rehabilitation and assistance^11–14^. Soft actuator based robots and devices have been studied in the recent past for biomedical rehabilitation and assistance applications including an assistive hand glove^11^, an artificial heart^13,15^ and artificial muscle packs^16^ that can potentially be implemented on various parts of the human body that may require assistance, such as the trunk carapace for gait assistance. Soft pneumatic actuators (SPA)^11,12,16–19^, fabricated using elastomeric silicones and typically composed of air corridors and chambers where input air pressure is applied, are particularly attractive for implementation in such robotic systems due to their ease and safety of operation, high power-to-weight ratio and low cost. Classical soft pneumatic actuators such as the McKibben actuators or pneumatic artificial muscles^20,21^ comprise of inflatable bladders wrapped inside braided mesh structures, where the motion of the actuator can be tuned by changing mesh patterns. Other soft actuator designs such as the PneuNets^18^ have multiple air chambers with narrow connecting passages and require multiple molding steps for fabrication. More recent actuator designs involve the actuators being fabricated in a single molding step, and reinforcement with fibers windings or stiffer shell structures to constrain any excessive inflation of the actuator body^12,19^. While a large majority of the currently existing actuator designs utilize positive air pressure input as the driving agent, recent developments have also demonstrated the successful implementation of soft actuators by reducing the pressure level inside elastomeric chambers to below that of atmospheric pressure (that is, to negative pressure, or partial vacuum) and achievement of compressive or buckling actuation motion profiles^22,23^.

The vacuum-driven soft pneumatic actuators (V-SPA) used to assemble the spinal column assistive device proposed and modeled in this work have an interconnected open-pore network and do not require additional molded air channels. The leading motivation for their development was to achieve simple actuation through the use of lightweight, readily available materials and minimal fabrication steps, to reduce mass and production effort. Their resulting simple composition and construction, consisting of foam encased with brushed-on silicone rubber skin, facilitates rapid development of customizable actuators for unique applications, or design iteration.

While soft materials such as foams and rubbers offer high agility and versatility, predicting the mechanical performance of the systems under consideration can be challenging due to the highly non-linear behavior of the materials used and the complex interaction between multiple materials employed. To capture more detailed information on stress-strain distributions within the actuators and model the non-linear effects observed more accurately, finite element analysis (FEA) of SPA has also been carried out in the past for several silicone-rubber based assistive soft systems for applications in hand rehabilitation^11^ and a prototype for a human heart^13^. Non-linear soft material behavior in silicone rubber based SPAs for a variety of different applications has been captured across a large range of realistic strains in^24^ using appropriate hyperelastic models. While silicone rubber based soft robotic systems have recently been well characterized, there is a lack of any study using FEA for SPA-based systems developed from alternate high-performance materials such as foams, targeted towards biomedical rehabilitation and biomechanical assistance. There is only a handful of examples in literature where soft robotic devices have been developed using foams^15,25^ and these studies have focused solely on experimental characterization, thereby lacking predictive capabilities for optimal system design.

In the present study, we present major findings from the development of a novel actuator design for an assistive device and its experimental results compared to predictions from the FEA. The presented actuator design, which forms the integral functional unit of the spinal column assistive device proposed, allows low-cost, manufacturable prototyping of functional modular units with highly versatile motion capabilities (e.g. both bending and linear motions achievable with the same module) and produces results in the desired performance range. The presented numerical models using FEA accurately predict the complex mechanical response and the performance obtained with the designed actuators, while allowing design iterations to optimize the critical functional parameters for the assistive system.

## Spine Assistance Module Design, Fabrication and Testing

**Figure 2:**
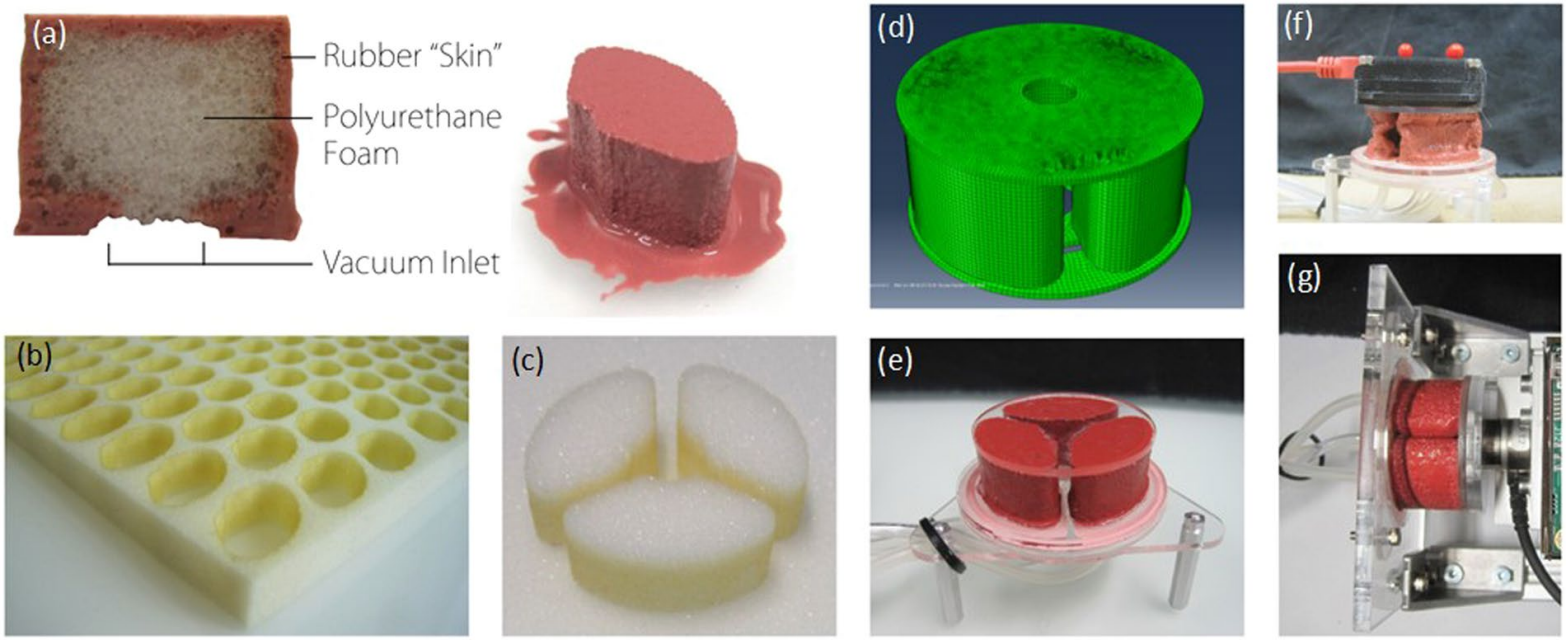
**(a**–**c)** Fabrication of the V-SPA module using a cutout polyurethane foam chamber which is painted with molten Elastosil rubber that acts as a restoring “skin” for the foam. Three such chambers are aligned as shown in (**c**) to form the 3-DOF module. **(d)** FE model for a single module containing the three chambers covered with top and bottom end-plates. **(e)** Fully assembled V-SPA module with top and bottom fiberglass end-plates. **(f)** A module undergoing free displacement testing, using an inertial measurement unit mounted on the top plate to measure displacements. **(g)** A module undergoing blocked force testing, using a load cell attached to the plate on the right side.

Current methods of soft pneumatic actuator fabrication^9^ commonly involve the casting of silicone rubbers into machined or 3D-printed molds which use either a sacrificial wax core or a two-part body construction to create hollow, inflatable structures. The necessary molds are time consuming to produce and cannot be reused following a change in actuator design. Without the need for such molds, V-SPA do not consume excess material or time greater than what is necessary for their own production. The technique used to fabricate the V-SPA presented in this work also ensures robustness and repeatability in manufacturing. The core component of the robotic module is a highly compressible actuation chamber, which forms the actuator body. To achieve large amounts of motion in compression, a light-weight, highly compressible polyurethane foam is used to form the base material for the chamber body. This foam core provides what is effectively an internal “mold” that can remain inside the actuator during use, while also providing the restoration force for actuator recovery following use. To strengthen the foam walls and achieve greater repeatability in overall mechanical performance of the V-SPA, the foam units are reinforced by painting the external surface of the foam walls (Fig. 2(a)) with a 0.5 mm thin coating of an elastomer which is an order of magnitude stiffer than the foam in the cured state. A schematic view of the actuator is shown in Fig. 1(b). The fabrication steps for the module are described in greater detail below.

A core in the shape of the final actuator is laser cut from an open-cell, polyurethane foam sheet (Fig. 2(b)). The cut-out shape is then mounted with glue to a small form, and coated with a layer of elastomeric polymer, to effectively form an air-sealed outer “skin”. Two coats are manually applied with a solid, flat, flexible brush onto the outer surface of the foam core to ensure complete coverage and sealing. To work properly, the chosen polymer must be compatible for curing in contact with the polyurethane foam, with adequate viscosity and surface tension properties to span the open cells in the outer surface of the foam core without entirely soaking into it. Silicone-based ELASTOSIL® M 4601 was found to be ideal for this purpose. Two other products tested, Ecoflex® 30 and Dragon Skin® 10, were not compatible with the foam tested. After a roughly 1 hour oven assisted curing time, the actuators can be used immediately. The glue form is removed with little relative force, enough to tear away a small portion of the foam surface to which it was attached, leaving an unsealed opening to which a vacuum supply line can be attached as an input to the actuator. When the resulting V-SPA is supplied with vacuum pressure, the flexible rubber outer skin is able to bend and collapse while the internal foam is easily crushed. The restoring force of both the outer skin and the internal foam act as a spring to return the actuator body to its original, unactuated form. The reinforcement skin coating on the compression chambers limits the level of buckling of the contained compressible material, so as to avoid excessive buckling and irreversible permanent deformation of the actuator body.

The use of foam as the fundamental physical element of V-SPA construction facilitates not only lightweight structure, but also their characteristic operational feature of utilizing vacuum as a primary power source. While conventional positive-pressure driven SPAs operate by converting inflation to a bending or linear profile through stretching of an elastic body, V-SPA operate as a result of the buckling and folding of a flexible structure induced by negative pressure. This difference affords two advantages for V-SPA in terms of efficiency as well as safety. The mode of deformation seen in V-SPA does not entail significant energy storage, which directly corresponds to a wastage of energy needed for activation, as well as a potential hazard in the case of sudden release upon actuator failure. Since the inflation of positive-pressure SPAs requires substantial elastic strain of its rubber structure, energy which is stored during activation can potentially discharge subsequent to a rupture, creating an abrupt and possibly dangerous impulsive shock to the system. In contrast, if a V-SPA is punctured during operation, it will simply cease to function, providing only the restoration force and passive stiffness of the remaining foam core which does not depend on maintaining internal vacuum. In addition, the possibility of failure caused by over-pressurization of typical SPAs is completely removed using vacuum as a power source because it is inherently limited by the environment from over-depressurization. Since the activation of a V-SPA depends on the reduction of its internal pressure, the greatest force and displacement which can be achieved will never exceed a bound imposed by the “maximum” input of zero pressure. This feature provides and additional layer of safety to vacuum-driven soft systems which serves to counter and protect against accidental or erroneous actuator commands, or the mechanical failure of subsystem components including valves and pressure regulators which could cause unintentional system behavior.

Various chamber structure patterns were compared and tested to evaluate the dependence of the mechanical performance of the actuator structure and the assistive device on the shape of the chamber. The chamber shape resulting in an optimal mechanical performance of the actuator structure for the assistive device is shown in Fig. 2(c). Rectangular, square or triangular cross-sectional shapes lead to larger stress concentrations due to sharp corners. Hence, those were not selected for further study. Circular cross-sectional shapes were also tested for the chambers and were found to yield larger mechanical instabilities in deformation response under compressive loading schemes. In addition to providing optimal mechanical strength, the chamber shape selected is also similar to the shape of the vertebrae comprising the human lumbar unit, leading to high conformance with the human body. The optimal height of the foam chambers was chosen in accordance with the transversal width, to provide adequate level of deformation during buckling, which is closely dependent on the slenderness ratio of the structure. The robotic module comprises of three such identical foam-skin V-SPA chambers, which are arranged at equal spacing as shown in Fig. 2(d,e). Selective pressurization and actuation of the chambers governs the pattern of displacement obtained with the actuator in the present study. Although it is possible to achieve other motion profiles by varying the arrangement and actuation pattern of the compression chambers, bending and linear motion were selected for further study in this work since these are sufficient to replicate and assist with the functionalities of the human lumbar unit.

Several experimental tests were done to characterize the performance of the actuators. These include performance in terms of free displacement, blocked force and blocked torque. The actuators were tested in free displacement to study the range of motion achieved as a function of the level of vacuum pressure applied. A venture principle vacuum ejector was used to generate the vacuum at different levels. To vary the level of vacuum used for actuation, the positive pressure input to the ejector was connected to a regulated pneumatic wall supply, varied manually using a flow control valve. By changing the input flow, the output vacuum flow and pressure was consequently varied. As this method did not allow repeatable setting of a desired vacuum pressure, instead the input supply was changed approximately and the actual resulting vacuum pressure on the output was measured directly with a pressure sensor (Honeywell, 24PCCFA6G). For these experiments, it was not important to achieve a particular pressure setting, it was only important to know what each pressure was for the sake of comparison. All measurements were then taken for a given pressure setting before changing to the next.

For these experiments, the bottom end-plate with the vacuum inlet was clamped in a rigid fixture. The top end-plate of the actuator was permitted to move freely while applying vacuum pressure, thereby generating a curved or straight trajectory, depending on how many chambers of the actuator were pressurized. To track the position of the actuator with a high speed camera, an inertial measurement unit (IMU) is mounted on the top end-plate, as shown in Fig. 2(f). To generate a curved trajectory, either one or two chambers of the actuator were pressurized. To generate a linear trajectory, all three chambers of the actuator were pressurized simultaneously at an equal level of vacuum pressure. After extracting the path for the actuator as a function of time, bending angles and linear displacement were characterized as a function of input vacuum pressure. The experiments were performed multiple times with each combination of chambers to analyze the positioning repeatability as well as the pressure-to-angle repeatability.

To test the maximum blocked force delivered by the linear actuators, each end-plate of the actuator was rigidly clamped, with the actuator in an un-pressurized state. On the distal clamped end of the actuator (i.e., the end opposite to the end with the air inlet), a six-axis force/torque sensor (as shown in Fig. 2(g)) was used to measure the force as well as the torque produced by the end-plate face of the actuator as the vacuum pressure was ramped up from zero. To characterize a single module, end-plates made from a relatively rigid material (Fiberglass, in this case) are glued to the top and bottom surfaces of the V-SPA assembly to facilitate attachment of fixtures for gripping the actuators during testing and ensure maximum force/torque delivery. To connect multiple modules for assembling and testing the assistive device proposed, interface PCB (Printed Circuit Board) layers, composed of FR4 fiberglass substrate coated in thin copper layers, were used. These boards serve the dual purpose of providing structural support for the mechanical interface and containing electrical circuits and components necessary for communication between modules and a central off-board controller. Additionally, the PCB layers contain outward-facing complementary male and female pin headers at the top and bottom, respectively, which are used for both joining actuator modules and sharing electrical signals and power.

## Mechanical Testing and Constitutive Material Model Fit

Computational modeling was done by using Finite Element Analysis (FEA) in ABAQUS/Standard (Simulia, Dassault Systems) to simulate the performance of the V-SPAs. 3-D models were created for the individual modules as well as for the entire assistive device. Models were developed to simulate both the linear and the bending modes of motion obtainable with each module. The details of the models are described below.

**Figure 3:**
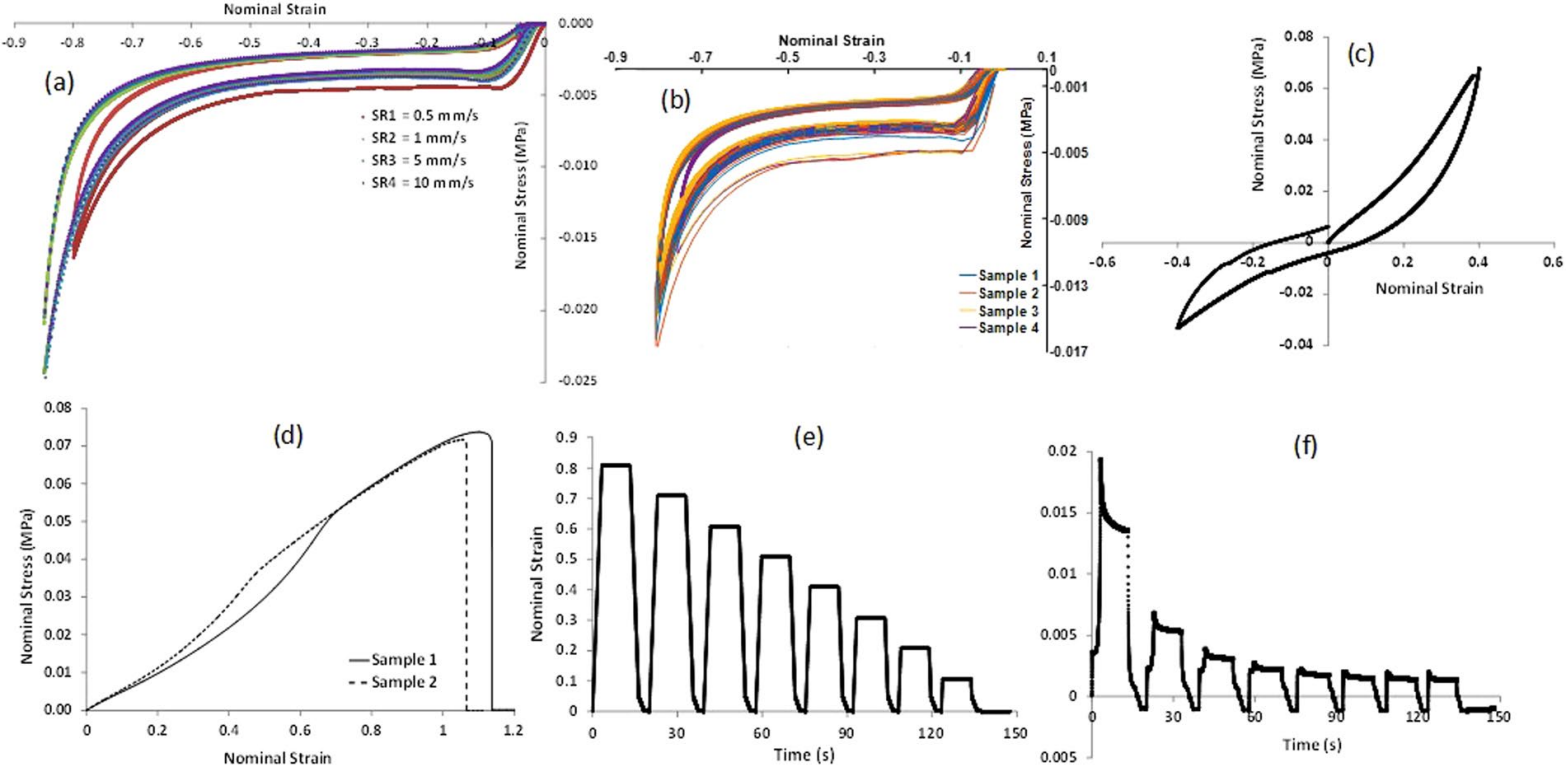
Stress-strain data for polyurethane foam samples undergoing mechanical testing under different modes of deformation. **(a)** Stress-strain data from uniaxial compression tests at different strain rates. The sample undergoes compression up to 80% of its original volume. **(b)** Cyclic test results in uniaxial compression. **(c)** Test results from simple shear testing. **(d)** Uniaxial tension test results. **(e**,**f)** Stress relaxation test results showing the decay of strain and stress vs. time, respectively.

To model the highly non-linear mechanical behavior of the foam actuator body, an appropriate constitutive model needs to be used. Two general constitutive laws which may be expected to produce reasonable results were evaluated, the Hyperfoam model and the Low Density Foam model. Hyperfoam materials are typically highly compressible. After testing compatibility with the material data, the model ultimately selected for the foam used to build the modules was the Hyperfoam model. The Abaqus Hyperfoam model is a nonlinear, isotropic material model that is valid for cellular solids with porosity that permits large volumetric changes, and is suitable for hyperelastic foams. Material properties were determined through multiple tests on small material samples, performed across a large range of relevant strains. The types of experimental data that are used for modeling foam materials include uniaxial, biaxial, planar, simple shear and volumetric. Since the actuator module under current study is primarily designed for loading in compression and bending, multiple cycles of uniaxial compression tests and simple shear tests were performed (shown in Fig. 3). The Hyperfoam model is defined by a strain energy potential function (*U*) in the form:

1 $$U({\hat{\lambda }}_{1},{\hat{\lambda }}_{2},{\hat{\lambda }}_{3})=\sum _{i=1}^{N}\frac{2{\mu }_{i}}{{\alpha }_{i}^{2}}[{\hat{\lambda }}_{1}^{{\alpha }_{i}}+{\hat{\lambda }}_{2}^{{\alpha }_{i}}+{\hat{\lambda }}_{3}^{{\alpha }_{i}}-3+\frac{1}{{\beta }_{i}}({({J}^{el})}^{-{\alpha }_{i}{\beta }_{i}}-1)]$$

The model can be used with any number of terms N, where *µ*, *α* and *β* are the primary fitting parameters. The independent variables λ_1_, λ_2_ and λ_3_ are the three principal stretches, and are related to the strain in a continuum. The term *J* ^*el*^ is the elastic volume ratio, and is a function of the principal stretches. The *β*_i_ parameters may be estimated directly from a given Poisson ratio, *ν*. The formulas for stress given strain can be determined for the different tests through differentiations of^1^ with the appropriate strain conditions. A material density of 0.0378 g/cm^3^, calculated experimentally using mass and volume measurements is assumed for the foam in the model.

In addition, time-dependent behavior of the foam within the duration of the loading is taken into account by incorporating viscoelastic effects into the model. To accomplish this, stress relaxation data is gathered to study the decay of the shear modulus as a function of time. To perform stress relaxation testing, a high tensile compressive strain is rapidly applied to a test specimen and held constant for a period of time, during which the stress is measured. The testing is carried out on a 20 mm cube sample of the foam. Cross-sectional area of sample is 400 mm^2^. Testing is carried out by compressing the sample upto 80% of its original volume, waiting for 10 seconds and observing the decay of stress at constant strain input (shown in Fig. 3(e,f)). Then the sample is taken back to its original position. This procedure is repeated multiple times. The decay of the stress is recorded as the material relaxes internally and then fit to the Prony series mathematical model in Abaqus. According to this model, the decay of the shear modulus (G) and the bulk modulus (K) as a function of time is expressed as:

2 $$G(t)={G}_{0}-\sum _{i=1}^{N}{G}_{i}[1-{e}^{(-t/{\tau }_{i}^{G})}]$$

3 $$K(t)={K}_{0}-\sum _{i=1}^{N}{K}_{i}[1-{e}^{(-t/{\tau }_{i}^{K})}]$$

where ${G}_{0}$ and ${K}_{0}$ are the instantaneous values of the moduli immediately after loading, ${G}_{i}$, ${K}_{i}$, ${\tau }_{i}^{G}$ and ${\tau }_{i}^{K}\,$are the fitting parameters and N is the number of summation terms for the fit. In this case, 13 terms were chosen in the Prony series so as to provide a good fit.

Since the foam-based actuator chambers are designed to undergo rapid deformations over multiple loading sequences, the foam material samples were also subjected to cyclic loading tests to understand and incorporate the stress-strain behavior of the samples over repetitive loading conditions. Permanent deformations were observed in the foam after loading for a few cycles. Thus, the samples were conditioned for 20 cycles before the stress-strain curve saturated to a repeatable level. The saturated stress-strain curves were then used as an input to the FE model to capture the module behavior post multiple cycles of loading and predict the long-term operation performance obtainable. Using the above described sets of experimental data as an input, the coefficients of the material model were calculated using the in-built functionality in Abaqus^TM^. These fitted coefficients for the material model were then directly used in the simulations without any further changes. The coefficients thus obtained are reported in Table 1 for the Hyperfoam model fit and in Table 2 for the Prony series fit.

**Table 1:** Hyperfoam Model Coefficients.

I	MU_I	ALPHA_I	NU_I
1	0.284002	13.2805	0.00000
2	−0.283367	13.9800	0.00000
3	4.680466E-07	−4.77281	0.00000

**Table 2:** Prony Series Fit Coefficients.

I	G_I	K_I	TAU_I
1	0.30030	0.30030	1.00140E-02
2	0.19970	0.19970	0.10016

To model the non-linear behavior of the reinforcement skin material (Elastosil), an incompressible, hyperelastic model was used. The Yeoh material model^26^, described by a strain energy function of the form $U=\,\sum _{i=1}^{N}{C}_{i}{({I}_{1}-3)}^{i}$ was used to describe the mechanical behavior of the skin. The material coefficients used for this model were *C*_1_ = 0.11 MPa, *C*_2_ = 0.02 MPa^19^. A material density of 1.07 g/cm^3^ is assumed for Elastosil in the model. Since the fabrication procedure for the modules involves painting the skin material onto the foam body, as described earlier in Section 2, it stays in contact with the foam at all instants of time. In the simulations, a tie constraint is imposed between the inner surface of the skin and the outer surface of the foam body along the entire circumference of the module to replicate this adhesive contact between the two interacting surfaces.

The Fiberglass material used to form the end-plates to grip the actuator modules in place during performance characterization was modeled using an elastic, brittle cracking model. A density of 1.9 g/cm^3^ was used in the model for this material. A Young’s modulus of 1770 MPa and Poisson’s ratio of 0.38 were used to describe the linear elastic range. Direct stresses after cracking and direct cracking strains were included in the model, with a cracking stress of 120 MPa at a cracking strain of 0.5. The end plates were attached to the top and bottom surfaces of the skin-foam assembly using tie constraints. The boundary conditions implemented on the plates depend on the type of test being carried out and are discussed in the next section. Finally, an external pressure was applied to all of the outer surfaces of the elastomeric skin, to model loading of the actuator module under vacuum. This external pressure level was varied to simulate varying levels of vacuum pressure that the actuator is subjected to during testing. Figure 2(d) shows the FE simulation model with three chambers.

**Figure 4:**
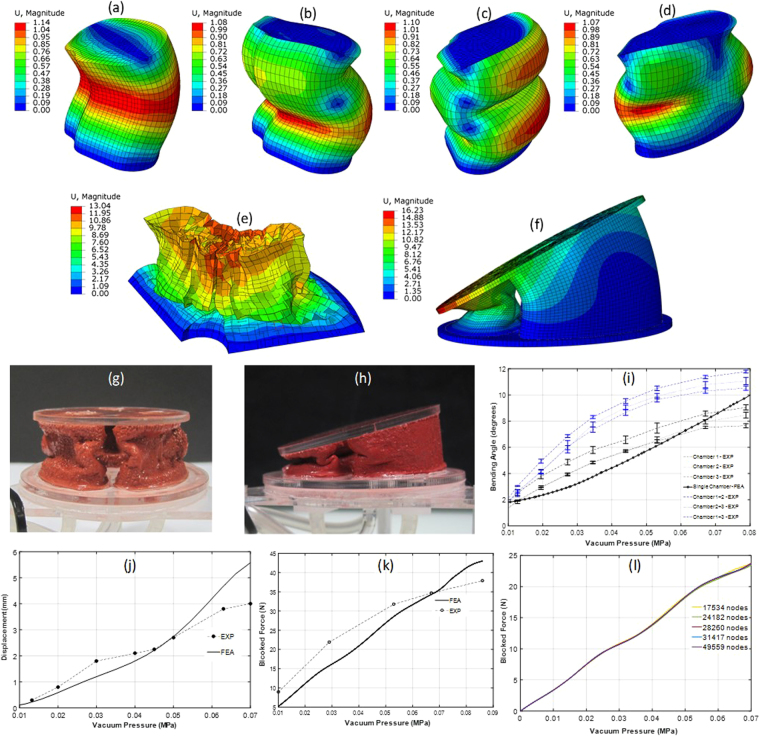
(**a**–**d)** Different eigenmodes of buckling for a single foam chamber subjected to compression loading. **(e)** Post-buckling collapse of a foam chamber using explicit analysis. **(f)** Free displacement simulation of the entire V-SPA module under bending conditions, with two foam chambers pressurized. **(g)** Image of the module exhibiting linear displacement profile with all chambers subjected to vacuum pressure. **(h)** Image of module exhibiting bending motion profile with two chambers subjected to vacuum pressure. **(i**–**k)** Comparison of simulation and experimental results for bending, linear displacement and blocked force tests, respectively. **(l)** Mesh convergence test results with varying total number of nodes in the system comprising the V-SPA module.

The actuator chambers are designed to undergo large mechanical deformations with buckling during pressurization. To model the buckling effects accurately, different solving schemes were tested to study the loading of the actuators. The implicit solving technique was first tested using an eigenmode superposition analysis with the Riks static method. This analysis was helpful in determining the critical buckling loading factor (load proportionality factor) as well as typical mode shapes expected (shown in Fig. 4(a–d)). Due to much larger solution times required to capture the dynamic behavior of the system using the Implicit method (as a result of the mandatory convergence checks enforced at each step and large, non-linear deformations involved in short time durations), ultimately an explicit solving scheme was utilized as the primary, robust solution method for all of the results presented in the subsequent section. The dynamic, explicit solver with a time period of 0.1 seconds and global minimum stable time increment estimator was used for the explicit solving scheme. The results obtained are presented and discussed in the next section.

## Single Module Characterization and Validation of Experimental Results

Experimental data is gathered for the single module actuators undergoing free displacement, in both linear and bending motion profiles, and blocked force testing, and compared to the simulation results. To characterize the actuators, the input vacuum pressure generated in the foam is decreased in steps from a maximum value of 78% to 0%, in steps of approximately 8–10%. Computationally, these tests are modeled using external pressure application, with the chamber face containing the vacuum supply inlet fixed in all directions. Figure 4(e–j) compare results from experiments and simulations for free displacement obtained as a function of input vacuum pressure. The pattern of deformation observed from the deformation contour plot for a single chamber obtained from simulations and shown in Fig. 4(e) is comparable to that observed in a single chamber in the experimental image shown in Fig. 4(g), for linear actuator motion. Similarly, for the bending mode, the deformation pattern obtained for a module from simulations and shown in the displacement contour plot in Fig. 4(f) is similar to the bending deformation shown in the corresponding experimental image in Fig. 4(h).

Tests are first performed for the modules undergoing free displacement in bending profile free displacement conditions and compared to the simulation results. The bending performance obtained is compared for the cases when only a single chamber is vacuum-pressurized versus when two chambers are pressurized. As expected, pressurizing two chambers leads to larger bending angles, although this difference is more pronounced at intermediate pressure values with more comparable bending angle values obtained at the highest pressures. The capability to tune the bending motion within the range shown in Fig. 4(i) demonstrates the large working space available with a single module and provides a great platform for building assistive devices that require high adaptability and flexibility. To simulate bending performance, the pressure load is applied only on the chambers that are subjected to vacuum, with all other surfaces being free to deform.

The contour plots for displacement obtained from simulations for bending performance, along with the matching images of the actuators at the corresponding values of input pressure are shown in Fig. 4. Figure 4(i) compares results from both experiments and simulations for bending free displacement obtained as a function of input pressure. At lower pressures, the simulations predict lower displacements, potentially due to the effects of residual permanent deformation in the modules over multiple cycles of testing and some preferred buckling modes. With further increase in pressure, simulation results catch up and rise slightly above the experiments at vacuum pressure levels of between 60–70%. A similar comparison is carried out for the actuators undergoing free displacement testing to obtain linear motion profiles. This is done by pressurizing all three chambers in the module at the same time, as opposed to selective chamber actuation for the bending case. The corresponding pressure load is applied in the FEA as well. Figure 4(j) compares results from both experiments and simulations for linear free displacement obtained as a function of input pressure. A similar trend is observed from the comparison in linear displacement as in the bending.

The blocked force tests for the modules are modeled with both the top and the bottom end-plate outward faces fixed in all directions (encastre), and applying the external pressure load with ramp increment. The net reaction forces generated at the nodes on the top end-plate face are summed up to obtain the blocked force generated. In the experimental configuration, the top and bottom end-plates are rigidly fixed with a load cell mounted on the top end-plate. All three chambers of the module are simultaneously pressurized at the same level to record the maximum force delivered by the module under linear compression and the measurements on the load-cell are recorded. Figure 4(k) compares results from experimental measurements and simulations for the blocked force testing.

Since the skin’s inner surface acts as the master surface and the foam’s outer surface is the slave surface in the contact between the skin and the foam, a smaller mesh element width is used to model the foam chambers as compared to the skin surfaces in all the cases modeled. A coarser mesh is used for the plates as the plate elements undergo smaller deformations than the foam and the skin. A uniformly sized mesh (i.e. no bias) is used in all the regions since there are no particular geometric stress concentration regions in the module. Mesh convergence testing effectively removed all mesh sensitivity from the analysis. The results are plotted in Fig. 4(l) and show the effect of mesh element size, along with the effect of changing the total number of nodes in the mesh for the entire system, on the blocked force obtained as a function of input vacuum pressure.

## V-SPA Module Assembly for Human Lumbar Unit and Spinal Cord Assistance

A case study of the biomechanics related to the human lumbar unit including the spinal cord is carried out in order to identify the design requirements and study the performance obtainable with the module assembly for potential application in assistive wearable systems. The primary modes of motion of the human spinal column include flexion-extension, lateral bending and twisting. Out of these different modes, the current 3-DOF actuator modules possess the capability to achieve flexion and lateral bending profiles with a large range of motion.

Flexion and lateral bending capabilities predicted from simulations and corresponding experimental measurements are described in further detail below. The results are first presented for the assembled assistive device created with the 3-chamber modules. While the 3-chambered module assembly possesses a large range of motion as a result of the resulting three DOF, a flatter and more compliant system interface surface would be required to conform to the human body to make the entire unit more wearable. Thus, simulation results are also presented for the proposed device comprised of 2-chamber modules which can be layered directly onto the vertebral portions of the back, so as to provide large forces and adequate conformity to the wearer’s spinal column.

**Figure 5:**
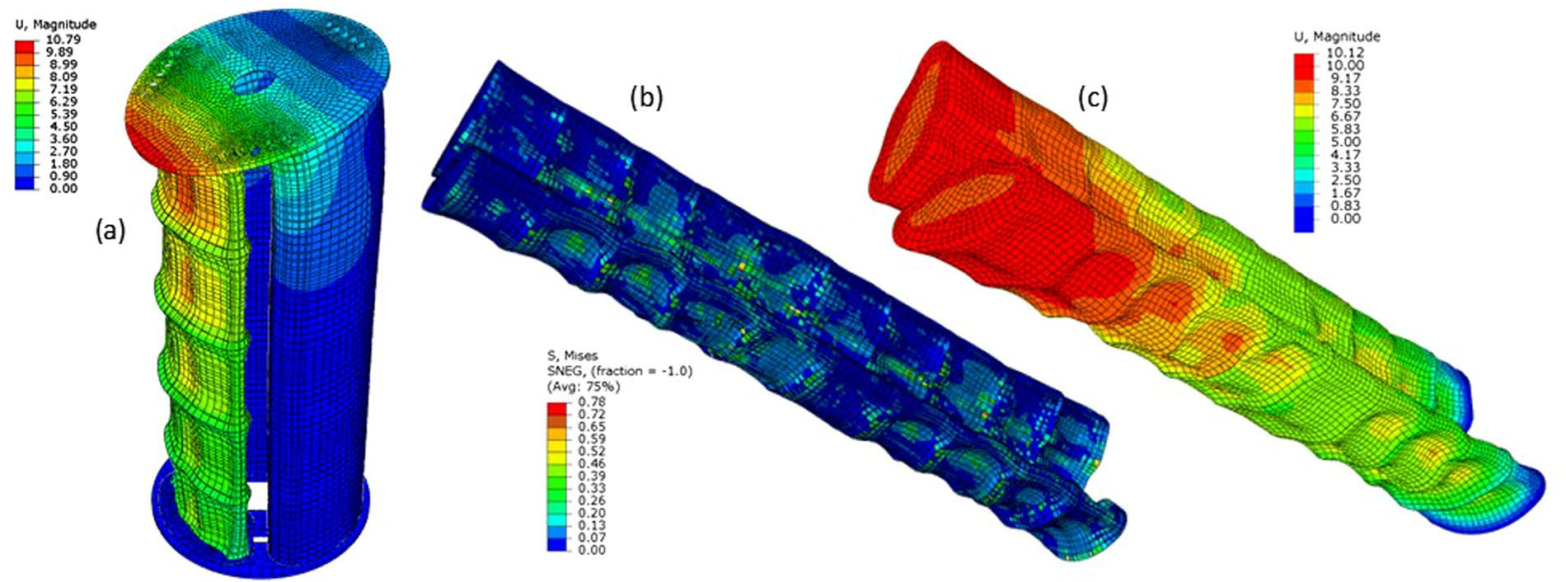
**(a)** Simulation result for a three column V-SPA module assembly in free displacement condition. The bottom plate is kept fixed. **(b)** Simulation result for a two column wearable V-SPA assembly made of the same structural units (comprising the foam core and Elastosil skin) as the three column assembly, in blocked ends condition, and under a distributed pressure loading scheme. The stresses generated in the assembly are closely representative of the stresses generated in the spinal column under high compression loading. **(c)** Displacement profile for the device under the same conditions as in (**b**).

Representative of the five vertebral sections comprising the lumbar unit, five identical V-SPA modules were connected in series and tested in free displacement and blocked conditions. The corresponding simulation results are obtained are shown in Fig. 5. Table 3 contains the experimentally recorded bending angles, blocked forces and blocked moments at the tip of the actuator modules placed at each level of the device forming the assembly representative of the lumbar unit, when the modules are pressurized through a common vacuum supply. The peak Von Mises stress data values recorded at any point within each module, obtained through numerical simulations, are also tabulated in Table 3. When the modules are connected in series, the pressure drops along the length of the assembly. The pressure level experienced at each individual module in unit is reported in Table 3. In the simulations, the progressive vacuum pressure drop along the length of the assembly is represented with the help of progressively decreasing pressure loads at each module.

**Table 3:** Design parameters for theV-SPA robotic assembly to enable spinal column assistance.

Location	Length (mm)	Vacuum Pressure (MPa)	Bending Angle (°)	Peak Mises Stress at any location (MPa)	Blocked Force at Tip (N)	Blocked Moment at Tip (N.mm)
First Module, L1	45	0.035	10	1.14	0.92	41.40
Second Module, L2	89	0.044	20	0.95	0.84	74.76
Third Module, L3	133	0.053	30	0.85	0.92	122.36
Fourth Module, L4	178	0.065	40	0.76	0.73	129.94
Fifth Module, L5	224	0.078	50	0.57	0.48	107.52

These results are then compared with FEA studies of the human spinal column. The pressurized V-SPA modules are capable of withstanding the peak Mises stresses within the range encountered by the spinal cord when subject to test compression forces in the ranges of 400–1200 N, as reported in^2^, implying their suitability for handling high compressive forces. The modules are also suitable for replicating or assisting with the first three vertebrae units and intervertebral discs. Additional structural stiffness would be required to replicate the set of mechanical functionalities of the fourth and the fifth lumbar units of the vertebrae under compressive loading conditions, since for these units the stress withstanding capabilities need to be double that of the first three units. This can potentially be achieved with minor changes such as covering the foam core with a thicker coating of the molten Elastosil or another elastomer with a higher stiffness. It should also be noted that the torque reported in Table 3 is at the very tip of the actuator where the top plate attached to the actuator is otherwise in a completely free configuration. Larger levels of torque may be achieved in other module configurations, such as the blocked-blocked configuration, where both the top and the bottom end-plates attached to the modules are fixed. Also, the torque obtained at the tip alone can be increased easily by coating the outer layer of the foam with a stiffer material or using a stiffer foam to form the core of the actuator, while keeping the overall design concept of the actuator the same.

In addition to adequate compression resistance, high flexion capability is desired at the same time. Due to the flexible foam core of the modules, the farthest V-SPA module can deliver a bending angle up to 50° in each DOF, as reported in Table 3. The lumbar spine is capable of generating a lateral bending motion of up to 35°. Thus, this range of motion is well within the capabilities of our proposed system. In forward movement, the lumbar spine can generate flexion of up to 75°. The presented five module V-SPA assembly can achieve up to 50° bending motion, but a larger range desired for forward flexion can be obtained by attaching more modules on top of the existing system. As observed from Table 3, the bending range of motion increases by 10° for each additional module attached. It is to be noted, however, that attaching more modules would potentially be at the expense of the compression withstanding capabilities of the system and that this would need to be carefully compensated for by increasing the stiffness of the system in strategic locations, so as to still deliver the desired range of motion.

## Discussion

Foam-based actuator modules provide an ideal platform for spinal cord assistance due to their inherent high compression and flexion capabilities, required to replicate the majority of the functionalities of the lower human spinal column. This can be potentially achieved with the help of the novel actuator design and predictive modeling capabilities presented in this work. Since the modules are designed to operate primarily under compression with the help of vacuum so as to enable energy optimization and easier manufacturability, extension motion is not supported in the system presented here but this could be enabled in future prototypes by integrating conventional SPAs (that extend linearly under the action of pressurized air input) into the module assembly alongside the V-SPAs. It is also possible to integrate twisting motion capability into the same V-SPA based platform by winding the elastosil outer skin layer with alternate sets of fiber reinforcements, similar to the concept demonstrated with SPAs in^27^.

The development of the assistive device proposed here is also motivated by the potential benefits of directly integrating actuation with other complimentary soft technologies. By utilizing a shared power supply, vacuum driven soft robots can also easily be enabled with stiffness tuning capabilities through layer or granular media “jamming”. Additionally, vacuum can be used to activate a variety of gripping or adhesive peripheral devices, including universal grippers for complex object manipulation and suction cups for inverted or inclined robot mobility. To enable these capabilities in addition to actuation on a common platform by conventional soft robotic standards would otherwise require separate power supply lines, with positive and negative pressures. Through the use of V-SPA and the corresponding fusion of independent pneumatic supply sub-systems, the complexity of multi-functional soft robots can be greatly reduced.

Although a detailed study on the interaction of the robotic module assembly proposed here with the human body and its implementation towards wearable assistance as well as portability is beyond the scope of the current work, the framework for such a device has been investigated through parallel studies. As with all wearable applications, key considerations for the development of a complete and operational device as proposed here include the careful selection of body attachment points, control strategies, and portability. For a lumbar region assistive device, it is envisioned that the active functional spinal elements would be integrated with a close-fitting vest or jacket-like base layer worn on the body, composed primarily of flexible fabrics and straps. To transfer forces and moments, specific areas of this device would be reinforced through stiff layers and braced against more prominent load carrying points on the body, particularly the hips and shoulders. For use outside of a fixed setting such as a workplace or clinic, the wearable spinal assistance apparatus may also be combined with a hip or back mounted portable computer control unit and pneumatic power supply, either capable of pressure generation or storage of a fixed charge of pressurized air. As this integration would increase the load on the body, the robotic lumbar support unit for assistance in these applications would require adequate scaling in design and performance to match and compensate for the additional load, and would be a subject of future study.

The actuator presented in this work is quite versatile due to its unique architecture and resulting motion capabilities. Furthermore, the actuators are easily manufactured and are scalable. The combined bending as well as linear motion achieved using the same actuator module platform, as shown in this work, holds promising potential for biomedical rehabilitation and wearable assistance not only for the discussed spinal column assistance but indeed for offering assistance in the form of artificial muscle pack/bundle- comprised soft exoskeletons for other body regions and limbs that may need motion assistance/restoration capabilities, such as the neck, hands or waist.

## Conclusions

Despite the availability of an adequate description of the biomechanics of the human spinal column, there is a lack of an existing solution to help restore the lost mechanical strength and mobility of the lumbar unit and the spinal cord while still enabling the overall mobility and high flexibility for the patient in the event of spinal cord injury. Soft actuators integrated into soft robotic systems hold great potential for implementation in such biomedical rehabilitation applications since they are capable of delivering flexible and safe motion profiles at low costs. Existing designs for soft pneumatic actuators suffer from limitations in manufacturability. Furthermore, conventionally used soft actuators and incorporated materials do not enable multi-mode locomotion capabilities and yield limited DOF. This work presents a new approach to design, fabricate and assemble vacuum driven soft actuators, along with computational models for these actuators, for application towards a spine assistive wearable device. This work presents the results obtained for actuator modules which deliver bending and linear displacement profiles using the same platform, depending on the actuator loading conditions. The motion profiles achieved with the modules are shown to replicate the functionalities of the human lumbar unit in forward flexion and lateral flexion movements.

Accurate and experimentally validated computational models have been developed using FEM for the actuator modules as well as for the assembled device. The FEM models enable the simulation of the actuator and device performance under a variety of different loading scenarios and provide predictions on their capabilities prior to fabrication. The stress concentration regions indicated in the simulations provide useful information regarding the design of the system and its failure regime. The findings from the models have been tested through experimental characterizations, enabling an understanding of the key factors influencing the performance of the proposed system.

## References

[1] Bunge RP, Puckett WR, Becerra JL, Marcillo A, Quencer RM (1993). Observations on the pathology of human spinal cord injury. A review and classification of 22 new cases with details from a case of chronic cord compression with extensive focal demyelination. Advances in Neurology..

[2] Ben-Hatira F, Saidane K, Mrabet A (2012). A finite element modeling of the human lumbar unit including the spinal cord. Journal of Biomedical Science and Engineering..

[3] Yoganandan N, Kumaresan S, Voo L, Pintar FA (1996). Finite element applications in human cervical spine modeling. Spine..

[4] Behrman AL, Harkema SJ (2000). Locomotor training after human spinal cord injury: a series of case studies. Physical therapy..

[5] Ilievski F, Mazzeo AD, Shepherd RF, Chen X, Whitesides GM (2011). Soft robotics for chemists. Angewandte Chemie..

[6] Shepherd RF (2011). Proceedings of the National Academy of Sciences. Multigait Soft Robot..

[7] Tolley MT (2014). A Resilient, Untethered Soft Robot. Soft Robotics..

[8] Lin H-T, Leisk GG, Trimmer B (2011). GoQBot: a caterpillar-inspired soft-bodied rolling robot. Bioinspiration & Biomimetics..

[9] Marchese AD, Katzschmann RK, Rus D (2015). A Recipe for Soft Fluidic Elastomer Robots. Soft Robotics..

[10] Cianchetti M (2014). Soft Robotics Technologies to Address Shortcomings in Today’s Minimally Invasive Surgery: The STIFF-FLOP Approach. Soft Robotics..

[11] Polygerinos P, Wang Z, Galloway KC, Wood RJ, Walsh CJ (2014). Soft robotic glove for combined assistance and at-home rehabilitation. Robotics and Autonomous Systems..

[12] Agarwal G, Besuchet N, Audergon B, Paik J (2016). Stretchable materials for robust soft actuators towards assistive wearable devices. Scientific Reports..

[13] Roche ET (2014). Actuators: A Bioinspired Soft Actuated Material. Advanced Materials..

[14] Asbeck AT, Schmidt K, Walsh CJ (2015). Soft exosuit for hip assistance. Robotics and Autonomous Systems..

[15] Mac Murray BC (2015). Poroelastic foams for simple fabrication of complex soft robots. Advanced Materials..

[16] Robertson MA, Sadeghi H, Florez JM, Paik J (2017). Soft pneumatic actuator fascicles for high force and reliability. Soft Robotics..

[17] Laschi C (2012). Soft robot arm inspired by the octopus. Advanced Robotics..

[18] Mosadegh B (2014). Pneumatic networks for soft robotics that actuate rapidly. Advanced Functional Materials..

[19] Polygerinos P (2015). Modeling of soft fiber-reinforced bending actuators. IEEE Transactions on Robotics..

[20] Tondu B (2012). Modelling of the McKibben artificial muscle: A review. Journal of Intelligent Material Systems and Structures..

[21] Chou C-P, Hannaford B (1996). Measurement and modeling of McKibben pneumatic artificial muscles. IEEE Transactions on Robotics and Automation..

[22] Yang, D., Verma, M.S., Lossner, E., Stothers, D. & Whitesides, G.M. Negative‐Pressure Soft Linear Actuator with a Mechanical Advantage. *Advanced Materials Technologies*. **2** (2017).

[23] Yang D (2015). Buckling of elastomeric beams enables actuation of soft machines. Advanced Materials..

[24] Moseley, P. *et al*. Modeling, design, and development of soft pneumatic actuators with finite element method. Adv. Eng. Mater. (2015).

[25] Argiolas A (2016). Sculpting Soft Machines. Soft Robotics..

[26] Yeoh O (1993). Some forms of the strain energy function for rubber. Rubber Chemistry and technology..

[27] Connolly F, Polygerinos P, Walsh CJ, Bertoldi K (2015). Mechanical programming of soft actuators by varying fiber angle. Soft Robotics..

