# Author Correction: Design and Computational Modeling of a Modular, Compliant Robotic Assembly for Human Lumbar Unit and Spinal Cord Assistance

**DOI:** 10.1038/s41598-018-31446-x

**Published:** 2018-08-30

**Authors:** Gunjan Agarwal, Matthew A. Robertson, Harshal Sonar, Jamie Paik

**Affiliations:** Ecole Polytechnique Federale de Lausanne (Swiss Federal Institute of Technology, EPFL) Reconfigurable Robotics Laboratory, EPFL-IGM-RRL, MED 11326, Station 9, CH-1015 Lausanne, Switzerland

Correction to: *Scientific Reports* 10.1038/s41598-017-14220-3, published online 31 October 2017

The V-SPA actuator that is the basis of this Article was originally reported in Reference 1, which is cited below. Further information about its mechanical component design, control and integration architecture, and physical experimental results for a diverse set of robots are detailed therein.
